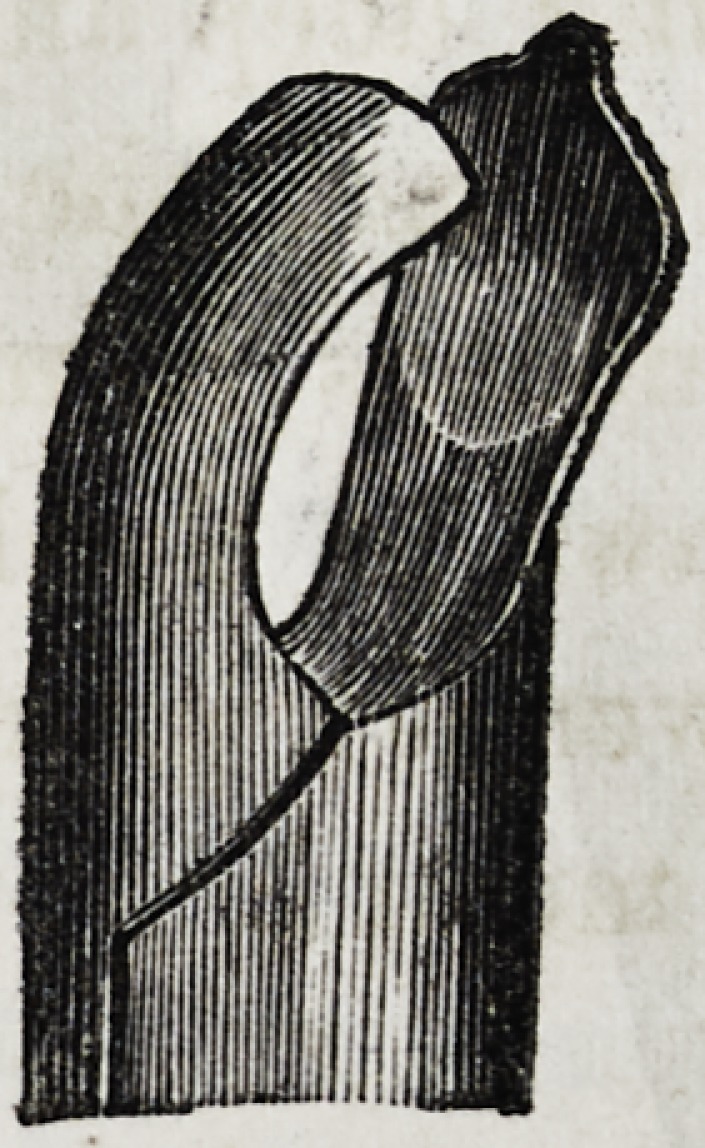# The Adapted Forceps

**Published:** 1857-07

**Authors:** James Robinson


### ARTICLE XIV.

### *The Adapted Forceps.*—By Mr. James Robinson.

No little discussion has, from time to time, taken place both in this country and America, as to who was the inventor of the improved forceps. Some of the disputants have asserted that they themselves were the inventors of the adapted instruments; while others have contended that these instruments were known, and in common use some sixty years back. Now, without at all attempting to settle this *vexatio quæstio*, I am disposed to hold that we owe much to Mr. Fay and Mr. P. Matthews, members of our profession, who, strangely enough, introduced the adapted forceps, at the same time; neither being aware of the other's improvement.

In the Transactions of the Society of Arts, vol. xliv, I find that Mr. Fay, having commented upon the imperfection of instruments then in use, goes on to say—

"I was soon convinced that the neck of the tooth was the only part on which the necessary force could be applied with the greatest safety and advantage. The teeth do not all present the same arrangements of parts; it follows that the figure of their neck must also vary. * * * There are certain teeth formed very much alike, both in the upper and lower jaw, and of the teeth thus similar to each other, there are various classes; but each class retains the same peculiarity of figure, in all ages and under all circumstances. * * * I have invented a set of instruments, accurately, because anatomically, suited to the several classes of teeth; a desideratum, as I believe, never before accomplished. These instruments are forceps corresponding in number to the different classes of teeth, and united to the same classes on the right side and on the left, in the upper and in the lawer jaw, amounting to six in number; but as it is necessary to have two or three sizes of these, the number of extracting forceps is nine. * * * The advantages which I consider these forceps possess over all others, are briefly these—First, they may be accurately applied to the necks of the several classes of teeth; they are made to fit the necks only, never making the least pressure on the enamel or body of the tooth; and, consequently, may be used without any danger of breaking a carious tooth in the attempt to extract it. Secondly, they never can slip when once accurately applied on the necks of the teeth—a great practical benefit. Thirdly, no cutting of the gum, or any other preparatory measure, is necessary, as the edges of the blades of the forceps may be at once brought upon the necks of the teeth. Fourthly, a provision is made by the beaked form of the extremities of the blades of the forceps, designed for the extraction of teeth having more than one fang, by which means the forceps may be steadily fixed on the remains of a decayed tooth, even when the edges of such teeth are below the level of the gum."

Such is the description, and the following are drawings, as given by Mr. Fay, of his very important invention:

I cannot agree with him in the mode of using his instruments, which he has explained; but I think we all owe him a debt of professional gratitude for his invention, and for the manner in which he directed the attention of the profession to the advantages of adapted forceps. Since he did so, very valuable improvements have been made in the adaptation of these instruments, as also in their manufacture. The late Mr. Shepard invented a forceps for the extraction of stumps, which also possesses great merit. Mr. Snell's improvement in the adaptation of the beaks of forceps to the upper and lower molar teeth, also deserves especial notice. This gentleman, it was, who suggested the curvature in a hook-like form of one of the handles. The object of this curve is to give the instrument such a hold on the little finger, as to prevent it from slipping whilst the operation is being performed.

In the construction of forceps, care should be taken that the surface of the blades are sufficiently expanded to grasp the neck of the tooth without pressure on the crown; and the forceps should be sufficiently large to afford the requisite purchase to the hand of the operator. For the removal of the temporary teeth, there should be six pairs, shorter in the handles and with smaller blades than those used for the permanent teeth. For the permanent teeth, twelve pairs are required; besides two pairs of stump forceps and two elevators. In addition to these, the pupil should be provided with a straight and a curved pair of bone forceps for the removal of splinters of the alveolar process.

This cut represents the forceps necessary for the extraction of the upper deciduous incisors. Only one pair of the above is necessary; but, for the extraction of the temporary molars, two pair are indispensable; one right, and the other left side, as

*Ibid.*

## Figures and Tables

**Figure f1:**
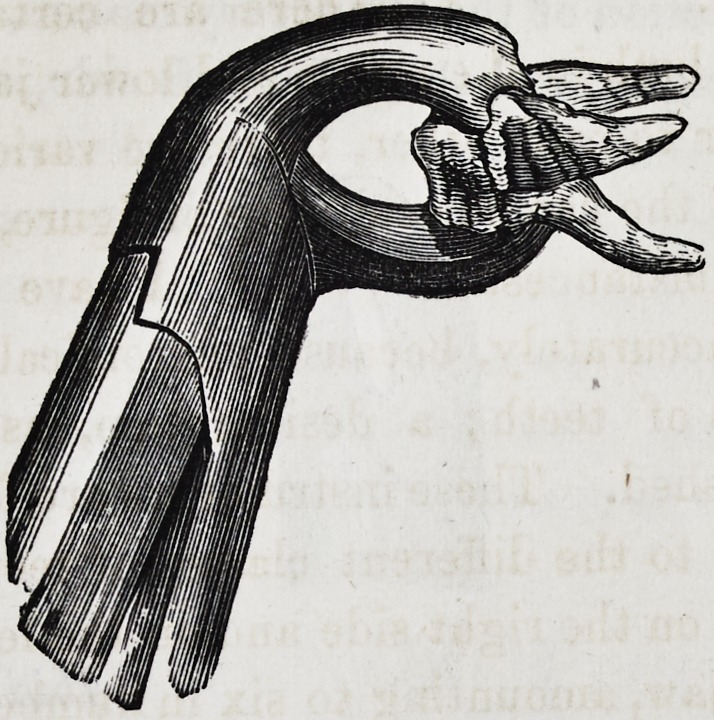


**Figure f2:**
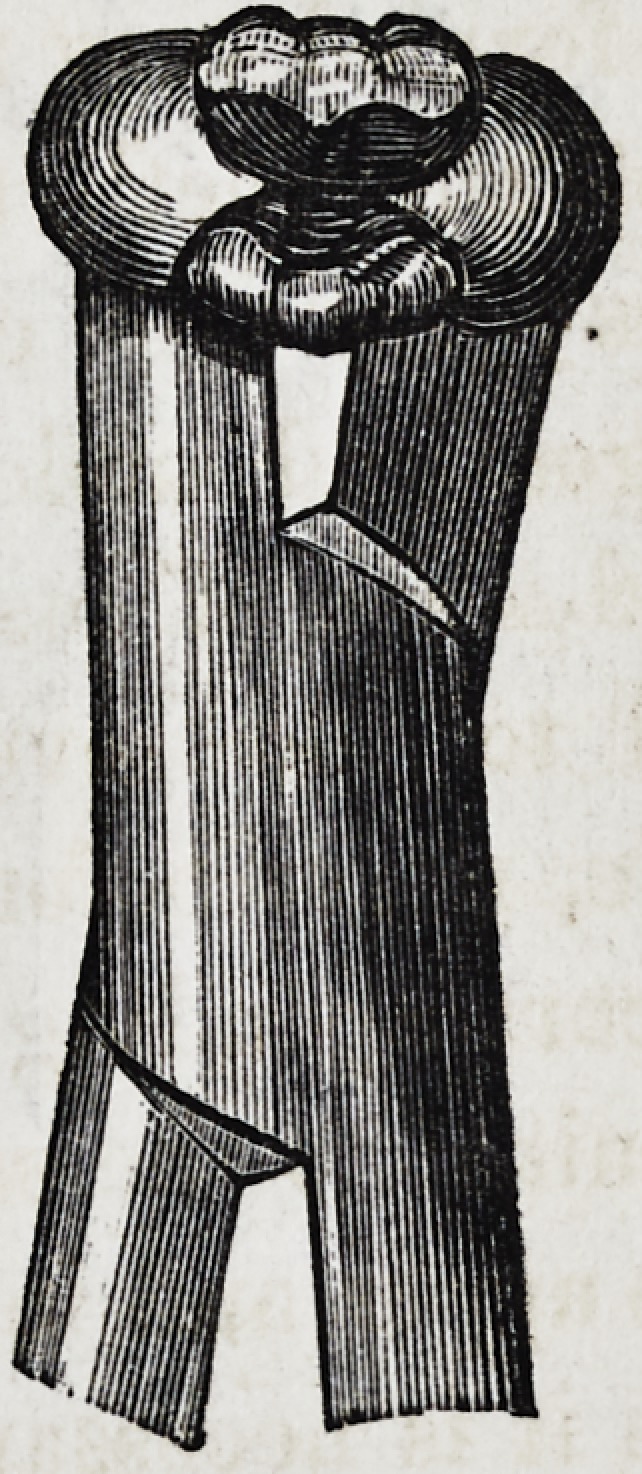


**Figure f3:**
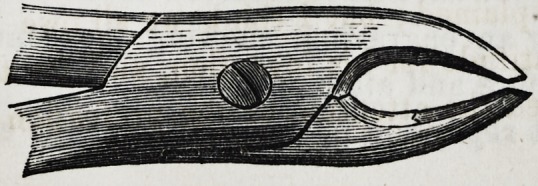


**Figure f4:**
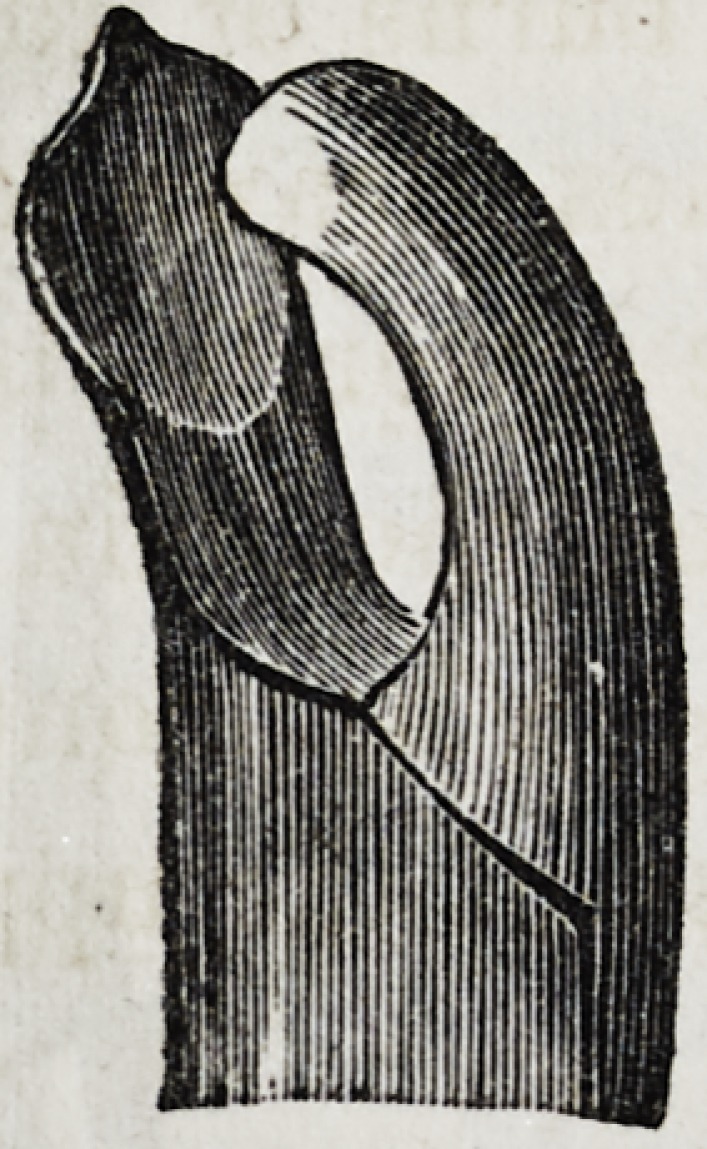


**Figure f5:**